# Health inequalities in the Netherlands: a cross-sectional study of the role of Type D (distressed) personality

**DOI:** 10.1186/1471-2458-12-46

**Published:** 2012-01-18

**Authors:** Marja JH van Bon-Martens, Johan Denollet, Lambertus ALM Kiemeney, Mariël Droomers, Monique JA de Beer, Ien AM van de Goor, Hans AM van Oers

**Affiliations:** 1 Academic Collaborative Centre Public Health Brabant, Tranzo, Tilburg School of Social and Behavioral Sciences, University of Tilburg, Tilburg, the Netherlands; 2 Department of Health Promotion, Regional Health Service Hart voor Brabant, 's-Hertogenbosch, the Netherlands; 3 CoRPS-Center of Research on Psychology in Somatic diseases, Department of Medical Psychology and Neuropsychology, Tilburg University, Tilburg, the Netherlands; 4 Department of Epidemiology, Biostatistics and HTA, Radboud University Nijmegen Medical Centre, Nijmegen, the Netherlands; 5 Centre for Prevention and Health Services Research, National Institute for Public Health and the Environment, Bilthoven, the Netherlands; 6 Department of Local Health Policy, Regional Health Service West-Brabant, Breda, the Netherlands; 7 Centre for Public Health Status and Forecasts, National Institute for Public Health and the Environment, Bilthoven, the Netherlands

## Abstract

**Background:**

In the Netherlands, as in many European countries, inequalities in health exist between people with a high and a low socioeconomic status (SES). From the perspective of the 'indirect selection hypothesis', this study was designed to expand our understanding of the role of Type D personality as an explanation of health inequalities.

**Methods:**

Data came from two cross-sectional Dutch surveys among the general population (aged between 19 and 64 years, response 53.7%, n = 12,090). We analyzed the relative risks of low SES, assessed using education and income, and Type D personality, assessed using the Type D Scale-14 (DS14), for different outcomes regarding lifestyle-related risk factors and health, using multivariate Generalized Linear Models.

**Results:**

Results showed that Type D personality was significantly associated with low SES (OR = 1.7 for both low education and low income). Moreover, the relative risks of Type D personality and low SES were significantly elevated for most adverse health outcomes, unconditionally as well as conditionally.

**Conclusion:**

The cross-sectional design hinders the making of definite etiological inferences. Nevertheless, our findings suggest that Type D personality does not explain the socioeconomic health inequalities, but is a risk factor in addition to low SES. Prevention of adverse health outcomes in low SES populations may have more effect when it takes into account that persons with a low SES in combination with a Type D personality are at highest risk.

## Background

In the Netherlands, as in many other European countries, inequalities in health exist between those of high and those of low socioeconomic status (SES) [1]. Life expectancy between the lowest and highest educated groups differs by 7.3 years for men and 6.4 years for women. Differences in healthy life expectancy are even larger, namely 19.2 years for men and 20.6 years for women [2]. Differences in (healthy) life expectancy between the lowest and highest income quintiles show the same pattern [3]. Moreover, a lower SES is associated with a higher prevalence of most chronic diseases, including mental disorders, self-assessed poor health, and lifestyle-related risk factors, such as current tobacco smoking and obesity [1,4,5]. Despite many efforts to reduce socioeconomic health inequalities in the Netherlands, most inequalities in health and lifestyle between educational levels remained unchanged [4-6].

Besides artefacts, such as measurement error, two major explanations for socioeconomic health inequalities have been proposed: causation and selection. Causation relates to causal mechanisms through which SES and social relationships potentially affect health status and the risk of dying. Selection or reverse causation refers to a set of pathways where unhealthy individuals may reduce their social position or become socially more isolated as a consequence of their inferior health status [7]. For selection, a distinction is made between direct selection, where a person's health status affects their social status, and indirect selection, meaning that some personal attributes, such as cognitive ability, coping styles, personality, and fitness, influence both the SES and the health of a person [7-10]. Several studies have shown that various personality traits partly explain the social gradients in mortality, health behaviour, and/or depression symptoms [11-15]. None of these studies, however, studied the role of the *distressed *or Type D personality.

In recent years, Type D personality was introduced in the cardiovascular literature as a valid and clinically relevant construct that has been associated with a three-fold increased risk of poor prognosis and morbidity in cardiac patients [16]. Type D personality refers to a general propensity to psychological distress that is defined by the combination of negative affectivity and social inhibition [17]. People who score high on negative affectivity have the tendency to experience negative emotions, while people who score high on social inhibition have the tendency to inhibit self-expression because of fear of disapproval by others. Persons with high levels on both personality traits are classified as having a Type D personality [17].

Given the clinical relevance of Type D personality in cardiovascular populations, it might also be of interest to assess the relevance of Type D personality for health risks and outcomes in the general population [18]. Following the 'indirect selection hypothesis', it was hypothesized that Type D personality would lead to both a lower SES and poorer health, thereby explaining (part of) the relationship between a lower SES and poorer health. This hypothesis was partly supported in a recent review of Type D studies in the general population, concluding that Type D personality is a vulnerability factor that may affect not only people with medical conditions, but also the health status of individuals from the general population [19]. However, the authors did not take SES into consideration. Type D personalities may deal with stress in a less adaptive way [20]. Type D personality is associated with major stressors such as traumatic events and social isolation, and with clinically significant burnout, depression and panic disorder [20,21]. These difficulties in dealing with stress might affect the upward social mobility or even increase the downward social mobility of Type D personalities. Moreover, the indirect selection mechanism might be explained by genetic factors that predispose for a Type D personality as well as for a low SES, for example through intelligence [9]. Therefore, the present study was designed to expand our understanding of the role of Type D personality as an explanation of health inequalities, with the aim of quantifying the contribution of Type D personality to the association between SES and different lifestyle-related risk factors and health.

## Methods

### Study design

This study used cross-sectional data from two surveys among the general population, collected by two Regional Health Services (RHSs) in the Netherlands to support local public health policy: one survey in the region West-Brabant (675,500 inhabitants at the time of the survey), and one survey in the municipality 's-Hertogenbosch, the capital city of the province Noord-Brabant (134,000 inhabitants at the time of the survey). RHSs in the Netherlands are authorised to sample the Municipal Basic Administrations (MBA; population register) for health surveys. For these two surveys, inhabitants aged between 19 and 64 years were randomly sampled from the MBA, stratified by municipality. The surveys were approved by the board of directors of the RHSs involved. According to the Dutch Medical Research Involving Human Subjects Act (WMO) these surveys were exempted from ethics approval because they did not meet the criterion that people are subjected to (invasive or bothersome) procedures or are required to follow rules of behaviour. Participants received a postal invitation to consent to participation by filling out an enclosed questionnaire, either on paper or, with a personal logon code, through the internet. The invitation also declared that the questionnaires would be processed anonymously. Data collection took place between October and December 2005. The initial sample for these two surveys consisted of 15,025 subjects, of whom 56.0% participated (n = 8,414) after a maximum of two reminders. In addition, 7,470 inhabitants were sampled non-representatively, for example in some deprived neighbourhoods or in some municipalities, with a response of 49.2% (n = 3,676).

### Main variables

#### Socioeconomic status

The dataset contained two indicators for SES: education and income. We defined low education as the case where the highest completed education is none or primary school, and low income as a net monthly household income below the Dutch standard (at the time of the study > €1,750.-).

#### Type D personality

The dataset contained the Type D Scale-14 (DS14), a short, easy-to-use, and valid construct, consisting of 14 questions about personality, with a 5-point Likert response scale ranging from 0 (false) to 4 (true). The DS14 comprises two subscales: the Negative Affectivity (NA) subscale and the Social Inhibition (SI) subscale. A pre-determined cut-off of ≥ 10 on both subscales was used to classify participants as Type D personality (i.e. NA of ≥ 10 and SI of ≥ 10) [17]. In the current dataset, the DS14 showed excellent internal consistency, with Cronbach's α = 0.87 for both subscales.

#### Lifestyle-related risk factors and health status

The dataset contained several variables as determinants of health (person-related factors, lifestyle, social and physical environment, prevention and care) and health status. The choice of the indicators used in this study was mainly based on the burden of disease in the Dutch population, leading to increased attention in Dutch health policy. For lifestyle-related risk factors, three indicators were used: (1) current tobacco smoking, (2) unsafe alcohol use, defined as the consumption of more than 21 glasses of alcoholic beverages weekly for men and more than 14 glasses weekly for women, and (3) obesity, defined as a body mass index of 30 or more [22]. For adverse health outcomes, five indicators were used: (1) self-assessed poor health, defined as fair or poor health based on the first question of the SF-36, (2) diagnosed by a physician as having one or more chronic illnesses on a list of eighteen, (3) diagnosed by a physician as having diabetes mellitus, (4) diagnosed by a physician as having cardiovascular disease (based on three questions: cerebrovascular accident or transient ischemic attack, myocardial infarct, and/or other severe heart disorder, such as heart failure or angina pectoris), and (5) high psychological distress (score of 30 or higher on the K10-version of Kessler Psychological Distress Scale) [23,24]. The K10 and its Dutch translated version have a good discrimination ability with respect to anxiety or depression disorders in the general population [23,25,26]. In our dataset, the K10 was available only for the municipality of 's-Hertogenbosch.

### Analysis

**Figure 1:**
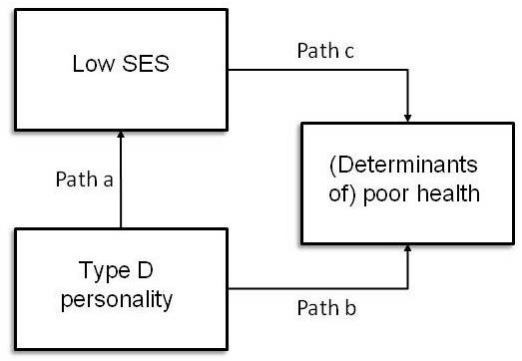
**Schematic model for indirect selection**.

Figure 1 presents our model of 'indirect selection' in a very simplified schematic way, for it ignores the bidirectional pathway (known as causation and direct selection) between low SES and health. Under the 'indirect selection hypothesis', Type D personality would be related to low SES (path a) as well as to (determinants of) health (path b). Moreover, under that hypothesis, an association between low SES and (determinants of) health (path c) would be (partly) explained by Type D personality. Yet it should be noted that, because of the cross-sectional nature of our data, the mechanisms of 'indirect selection' and 'causation' cannot be distinguished. The abovementioned associations could also occur in the case of causation when a lower SES would be associated with both a type D personality and poorer health, while at the same time type D personality would be related to poorer health. The following associations were assessed and quantified from the perspective of 'indirect selection', all adjusted for age, sex, and municipality:

1. the association between Type D personality and a low SES (path a);

2. the association between Type D personality and (determinants of) health (path b);

3. the association between low SES and (determinants of) health (path c);

4. the association between low SES and (determinants of) health, conditional on Type D personality (path c, controlled for path a and path b); and

5. modification of the effect of low SES on (determinants of) health by Type D personality (interaction).

For the first analysis, we computed the odds ratios with 95% confidence intervals for low SES as a function of Type D personality, using logistic regression analysis. For the second, third, and fourth analyses, we computed relative risks with 95% confidence intervals for Type D personality (2) and low SES (3 and 4) as risk factors for (determinants) of health, using multivariate Generalized Linear Models. In addition, this relative risk for low SES was adjusted for Type D personality in the fourth analysis. In all these analyses, each reference category contained all persons without the studied characteristic. For the fifth analysis, a new variable was constructed for all four possible response combinations of Type D personality and low SES. Using as the reference category the category where both Type D personality and low SES were absent, we computed relative risks with 95% confidence intervals for the other three combinations of Type D personality and low SES as risk factors for (determinants) of health, using multivariate Generalized Linear Models. We computed the Relative Excess Risk due to Interaction (RERI) in order to assess and quantify interaction on an additive scale, as suggested by Rothman [27]. The 95% confidence intervals for the RERIs were computed with a bootstrapping procedure, with a sample size of 10,000, using Knol's bootstrapping script, adjusted for R-Plus [28]. The covariates sex, age, and municipality were taken into consideration for all associations.

## Results

Table 1 presents the prevalence of the main variables in both initial samples (n = 8,414), after weighting for sex, age, and municipality, according to the demographics of the populations. Type D personality was found in one fifth of both populations. Social inhibition occurred more often than negative affectivity, especially in the West-Brabant region. Low education (highest completed education none or primary school) was less prevalent (6.8-7.5%) than a low income (income below Dutch standard; 38.3-41.5%).

**Table 1:** Weighted prevalences of the main variables

Characteristic	Region	
	**West-Brabant**	**'s-Hertogenbosch**

	**(n = 7,764)**	**(n = 650)**

	**%**	**%**

Sex		

Male	50.5	50.3

Age		

19-34 years	30.5	33.6

35-49 years	37.7	37.7

50-64 years	31.8	28.6

Type D personality (DS-14)		

Negative affectivity (NA ≥ 10)	31.7	31.8

Social inhibition (SI ≥ 10)	40.5	34.5

Type D personality (NA ≥ 10 and SI ≥ 10)	20.4	19.1

Highest completed education		

None or primary school	7.5	6.8

Lower general secondary or lower vocational school	35.2	30.7

Higher general secondary school, intermediate vocational school, or pre-university	33.4	28.1

Higher vocational (Bachelor) or university (Master)	23.9	34.3

Net monthly household income		

≤ € 850	8.9	9.6

€851-€1,150	8.4	11.8

€1,150-€1,750	21.0	20.1

€1,751-€3,050	28.2	25.9

€3,051-€3,500	6.7	9.1

≥ €3,501	8.4	9.7

Doesn't want to tell	18.4	13.7

Tobacco smoking		

Current	31.3	34.7

Former	31.5	28.0

Never	37.2	37.3

Alcohol consumption		

Unsafe^a^	12.1	12.2

Safe	66.1	66.2

Abstains	14.6	13.7

Yes, amount unknown	7.2	7.9

Body Mass Index		

< 30 kg/m^2^	89.2	88.6

≥ 30 kg/m^2^	10.8	11.4

Self-assessed health		

Excellent	6.8	8.2

Very good	20.7	22.7

Good	59.8	55.3

Fair	11.2	12.3

Poor	1.5	1.5

At least one chronic disease, diagnosed by physician^b^		

Yes	40.2	40.7

No	59.8	59.3

Diabetes		

Yes, diagnosed by physician	3.4	3.4

No/not diagnosed by physician	96.6	96.6

Cardiovascular disease^c^		

Yes, diagnosed by physician	2.5	2.9

No/not diagnosed by physician	97.5	97.1

Psychological distress		

None or low (K10 score 10-15)	-	66.5

Moderate (K10 score 16-29)	-	28.6

High (K10 score 30-50)	-	4.9

^a ^> 21 glasses of alcoholic beverages weekly for men; > 14 glasses of alcoholic beverages weekly for women

^b ^during the last 12 months, from among the following 18 chronic diseases: 1) diabetes; 2) stroke, cerebrovascular accident or transient ischemic attack; 3) myocardial infarction; 4) other severe heart disorder, such as heart failure or angina pectoris; 5) cancer; 6) migraine or regular severe headaches; 7) high blood pressure; 8) constriction of the blood vessels in stomach or legs (not varicose veins); 9) asthma, chronic bronchitis, pulmonary emphysema, or COPD; 10) severe or persistent intestinal disorders, for more than 3 months; 11) psoriasis; 12) chronic eczema; 13) incontinence; 14) severe or persistent back disorders (including slipped disc); 15) articular degeneration of hips or knees; 16) chronic arthritis (rheumatoid arthritis, chronic rheumatism); 17) other severe or persistent disorder of neck or shoulder; 18) other severe or persistent disorder of elbow, wrist, or hand

^c ^based on three questions: (1) stroke, cerebrovascular accident, or transient ischemic attack, (2) myocardial infarction, (3) other severe heart disorder, such as heart failure of angina pectoris

Examination of the occurrence of Type D personality over the categories of SES and (determinants of) health showed that the prevalence of Type D personality increased with decreasing education and income (Table 2). With regard to lifestyle-related risk factors, the most striking finding was the highest prevalence of Type D personality in the alcohol abstainers. As to health, there seemed to be a dose-response relationship between, on the one hand, self-assessed health and psychological distress, and, on the other hand, the prevalence of Type D personality: the poorer the self-assessed health or the higher the psychological distress, the higher the prevalence of Type D personality (Table 2).

**Table 2:** Weighted prevalence of Type D personality

Characteristic	Region	
	**West-Brabant**	**'s-Hertogenbosch**

	**% Type D**	**% Type D**

Highest completed education		

None or primary school	31.0	34.1

Lower general secondary or lower vocational school	23.6	20.3

Higher general secondary school, intermediate vocational school, or pre-university	19.0	20.8

Higher vocational (Bachelor) or university (Master)	14.4	14.1

Net monthly household income		

≤ € 850	32.8	23,3

€851-€1,150	30.1	24.0

€1,150-€1,750	21.8	21.4

€1,751-€3,050	17.6	21.6

€3,051-€3,500	10.9	12.3

≥ €3,501	13.3	11.5

Doesn't want to tell	19.3	14.0

Tobacco smoking		

Current	23.9	17.9

Former	19.1	21.8

Never	18.7	17.9

Alcohol consumption		

Unsafe^a^	16.3	8.9

Safe	19.1	18.2

Abstains	29.3	32.6

Yes, amount unknown	21.7	18.4

Body Mass Index		

< 30 kg/m^2^	19.9	18.0

≥ 30 kg/m^2^	24.1	26.8

Self-assessed health		

Excellent	5.4	3.8

Very good	9.7	13.0

Good	20.5	17.8

Fair	44.0	43.0

Poor	55.7	50.0

At least one chronic disease^b^		

Yes, diagnosed by physician	23.4	21.9

No/not diagnosed by physician	17.6	15.6

Diabetes		

Yes, diagnosed by physician	19.7	18.2

No/not diagnosed by physician	20.2	18.7

Cardiovascular disease^c^		

Yes, diagnosed by physician	23.4	16.7

No/not diagnosed by physician	20.2	18.5

Psychological distress		

None or low (K10 score 10-15)		7.8

Moderate (K10 score 16-29)		37.7

High (K10 score 30-50)		64.5

a > 21 glasses alcoholic beverages weekly for men; > 14 glasses alcoholic beverages weekly for women

b during the last 12 months, from among the following 18 chronic diseases: 1) diabetes; 2) stroke, cerebrovascular accident or transient ischemic attack; 3) myocardial infarction; 4) other severe heart disorder, such as heart failure or angina pectoris; 5) cancer; 6) migraine or regular severe headaches; 7) high blood pressure; 8) constriction of the blood vessels in stomach or legs (not varicose veins); 9) asthma, chronic bronchitis, pulmonary emphysema, or COPD; 10) severe or persistent intestinal disorders, for than 3 months; 11) psoriasis; 12) chronic eczema; 13) incontinence; 14) severe or persistent back disorders (including slipped disc); 15) articular degeneration of hips or knees; 16) chronic arthritis (rheumatoid arthritis, chronic rheumatism); 17) other severe or persistent disorder of neck or shoulder; 18) other severe or persistent disorder of elbow, wrist, or hand

c based on three questions: (1) stroke, cerebrovascular accident, or transient ischemic attack, (2) myocardial infarction, (3) other severe heart disorder, such as heart failure of angina pectoris

Using the total dataset (n = 12,090), adjusted for sex, age, and municipality, Type D personality was significantly associated with both indicators of a low SES: low education (ORadj = 1.7, 95%CI: 1.5-2.0) and low income (ORadj = 1.7, 95%CI: 1.6-1.9) (not tabulated).

Persons with a Type D personality had a small but significantly higher risk of current tobacco smoking (RRadj = 1.1, 95% CI: 1.1-1.2), but not of unsafe alcohol use and obesity (Table 3). Furthermore, Type D personalities were at a higher risk of self-assessed poor health (RRadj = 2.8; 95% CI = 2.6-3.1), chronic disease (RRadj = 1.2, 95% CI = 1.1-1.2), cardiovascular disease (RRadj = 1.6, 95% CI = 1.2-2.0), and high psychological distress (RRadj = 8.6, 95% CI = 4.9-15.1). Type D personalities did not have an elevated risk of diabetes. The associations all remained statistically significant when they were analyzed conditionally on low education or on low income, though some relative risks moved slightly towards the null value (Table 3).

**Table 3:** Results for the (un)conditional associations^a ^of Type D personality and low SES

	Outcome is a lifestyle-related risk factor			Outcome is poor health				
	**Current tobacco smoking**	**Unsafe alcohol use**	**Obesity**	**Self-assessed poor health**	**Chronic disease**	**Diabetes**	**Cardiovascular disease**	**High psychological distress**

	**RRadj^b^** **(95% CI)**	**RRadj^b^** **(95% CI)**	**RRadj^b^** **(95% CI)**	**RRadj^b^** **(95% CI)**	**RRadj^b^** **(95% CI)**	**RRadj^b^** **(95% CI)**	**RRadj^b^** **(95% CI)**	**RRadj^b^** **(95% CI)**

Type D personality								

Unconditional	1.1* (1.1-1.2)	0.9 (0.8-1.0)	1.1 (1.0-1.3)	2.8* (2.6-3.1)	1.2* (1.1-1.2)	1.0 (0.7-1.2)	1.6* (1.2-2.0)	8.6* (4.9-15.1)

Conditional on low education	1.1* (1.0-1.2)	0.9 (0.8-1.0)	1.1 (1.0-1.2)	2.6* (2.4-2.8)	1.2* (1.1-1.2)	0.9 (0.7-1.2)	1.5* (1.2-1.9)	7.9* (4.5-13.9)

Conditional on low income	1.1* (1.0-1.2)	0.9 (0.8-1.0)	1.1 (1.0-1.2)	2.5* (2.3-2.8)	1.2* (1.1-1.2)	0.9 (0.7-1.1)	1.4* (1.1-1.8)	7.3* (4.2-12.9)

Low education								

Unconditional	1.4* (1.3-1.5)	0.8* (0.6-0.9)	1.7* (1.4-1.9)	2.4* (2.2-2.7)	1.2* (1.1-1.3)	1.8* (1.4-2.3)	1.9* (1.5-2.5)	3.9* (2.2-7.1)

Conditional on Type D personality	1.4* (1.3-1.5)	0.8* (0.6-0.9)	1.6* (1.4-1.9)	2.1* (1.9-2.3)	1.2* (1.1-1.3)	1.8* (1.4-2.3)	1.9* (1.4-2.5)	2.9* (1.7-4.9)

Low income								

Unconditional	1.4* (1.3-1.5)	1.1 (1.0-1.2)	1.2* (1.1-1.4)	2.2* (2.0-2.5)	1.2* (1.1-1.2)	1.6* (1.3-2.0)	1.9* (1.6-2.4)	3.8* (2.1-6.9)

Conditional on Type D personality	1.4* (1.3-1.5)	1.1 (1.0-1.2)	1.2* (1.1-1.3)	2.0* (1.8-2.2)	1.1* (1.1-1.2)	1.6* (1.3-2.0)	1.8* (1.5-2.3)	2.9* (1.6-5.2)

^a ^Using multivariate Generalized Linear Models

^b ^Adjusted for sex, age (three categories), and municipality

* *p *< 0.05

Persons with low education as well as those with a low income had significantly higher relative risks for all studied indicators for (determinants of) health, except for unsafe alcohol use (Table 3). The risk of unsafe alcohol use was significantly lower for persons with a low education (RRadj = 0.8, 95% CI: 0.6-0.9). All associations remained statistically significant when they were analyzed conditionally on Type D personality, though some relative risks moved slightly towards the null value (Table 3).

Interaction between Type D personality and low SES on an additive scale was significant for the effect of low education on high psychological distress (RERI = 12.9, 95% CI: 0.8-32.3), and for the effect of a low income on self-assessed poor health (RERI = 1.4, 95% CI: 0.9-1.9) and on high psychological distress (RERI = 11.4, 95% CI: 3.5-41.0) (Table 4). This means, for example, that the relative risk for self-assessed poor health is 1.4 higher in Type D personalities with a low income than if there were no interaction between Type D personality and low income. Because the absolute background risk was 5.8% (the prevalence of a poor self assessed health in the absence of a Type D personality and a low income in the region West-Brabant) this means that the absolute excess risk due to interaction is 8.1% (5.8% × 1.4). Accordingly, the excess risk due to interaction for high psychological distress is 27.1% (2.1% × 12.9) for the interaction between Type D personality and low education and 8.0% (0.7% × 11.4) for the interaction between Type D personality and low income, based on the background prevalence in the region 's-Hertogenbosch.

**Table 4:** Results for the modification of effects^a ^of Type D personality and low SES

	Outcome is a lifestyle-related risk factor			Outcome is poor health				
	**Current tobacco smoking**	**Unsafe alcohol use**	**Obesity**	**Self-assessed poor health**	**Chronic disease**	**Diabetes**	**Cardiovascular disease**	**High psychological distress**

	**RRadj^b^** **(95% CI)**	**RRadj^b^** **(95% CI)**	**RRadj^b^** **(95% CI)**	**RRadj^b^** **(95% CI)**	**RRadj^b^** **(95% CI)**	**RRadj^b^** **(95% CI)**	**RRadj^b^** **(95% CI)**	**RRadj^b^** **(95% CI)**

Type D Personality and low education								

Both absent	1	1	1	1	1	1	1	1

Only low education	1.4* (1.3-1.5)	0.8* (0.6-1.0)	1.8* (1.5-2.1)	2.6* (2.3-3.1)	1.2* (1.1-1.3)	1.8* (1.3-2.3)	2.0* (1.5-2.8)	3.2 (0.9-10.9)

Only Type D	1.1* (1.0-1.2)	0.9 (0.8-1.1)	1.1* (1.0-1.3)	2.9* (2.6-3.2)	1.2* (1.1-1.3)	0.9 (0.7-1.2)	1.6* (1.2-2.1)	8.1* (4.3-15.3)

Both present	1.5* (1.3-1.8)	0.7 (0.5-1.0)	1.5* (1.2-1.9)	5.1* (4.5-5.9)	1.4* (1.2-1.5)	1.9* (1.2-2.9)	2.6* (1.7-4.1)	23.1* (11.4-46.9)

RERI^c^	0.0 (-0.2-0.3)	0.0 (-0.4-0.3)	-0.4 (-0.9-0.1)	0.6 (-0.1-1.3)	0.0 (-0.2-0.1)	0.2 (-0.7-1.1)	0.0 (-1.2-1.4)	12.9* (0.8-32.3)

Type D Personality and low income								

Both absent	1	1	1	1	1	1	1	1

Only low income	1.4* (1.3-1.4)	1.1 (1.0-1.2)	1.2* (1.1-1.4)	2.0* (1.8-2.3)	1.1* (1.1-1.2)	1.6* (1.3-1.9)	1.8* (1.4-2.3)	2.2 (0.8-6.1)

Only Type D	1.0 (0.9-1.1)	0.9 (0.8-1.1)	1.1 (1.0-1.3)	2.6* (2.3-3.1)	1.1* (1.1-1.2)	0.8 (0.5-1.2)	1.4 (1.0-2.1)	5.6* (1.9-16.2)

Both present	1.5* (1.4-1.7)	0.9 (0.8-1.1)	1.3* (1.1-1.5)	5.1* (4.5-5.7)	1.4* (1.3-1.4)	1.5* (1.1-2.1)	2.7* (2.0-3.6)	18.2* (7.8-42.7)

RERI^c^	0.2 (0.0-0.3)	-0.1 (-0.3-0.2)	-0.1 (-0.3-0.2)	1.4* (0.9-1.9)	0.1 (0.0-0.2)	0.2 (-0.4-0.7)	0.4 (-0.5-1.3)	11.4* (3.5-41.0)

^a ^Using multivariate Generalized Linear Models

^b ^Adjusted for sex, age (three categories), and municipality

^c ^RERI: Relative Excess Risk due to Interaction

* *p *< 0.05

## Discussion

Some methodological limitations should be considered when interpreting the results of our study. First, due to the cross-sectional nature of the datasets, it is not possible to make any definite inference on causality. However, we assumed that both Type D personality and a low SES precede the outcomes for (determinants of) health. Second, the response of Type D personalities, persons with a lower SES, and those with poor health could be lower than that of others. Selective non-response of these persons would lead to underestimation of their prevalence, and would lead to underestimation of the real risk ratios only when Type D personalities and/or persons with a lower SES did not respond in the presence of (determinants of) poor health. Third, Type D personalities might respond differently to particular questions. For example, Type D personalities are inclined to perceive poor health more often than non Type D personalities [29]. Socioeconomic differences in 'life expectancy in good health' might partly be explained by this inclination, because this outcome is based on self-assessed health combined with mortality. Moreover, in the presence of health complaints, Type D personalities are less likely to consult a physician as compared to non Type D personalities for physical or mental health problems [30-32]. This could result in under diagnosis and, consequently, underestimation of real risk ratios of chronic diseases. Fourth, for the reason of comprehensiveness, we've chosen to dichotomize the measures of SES, which could have been used as ordinal variables in the analysis. Therefore, due to measurement imprecision and loss of data, our associations were measured more conservatively than by using ordinal variables, possibly leading to underestimation of the association and interaction measures. Fifth, we did not select some covariates that might be relevant, particularly ethnicity. For example, non-Western respondents in the West-Brabant region more often had low education (28%) and a low income (74%) than Western respondents (6% and 36% respectively). In addition, among the non-Western respondents in this region, the prevalence of Type D personality was much higher (33%) than among Western respondents (19%). Therefore, we repeated our analyses on the subset of Western respondents, and that showed that most of the results remained essentially unchanged. Sixth, some questions of the K10 to assess psychological distress seem to overlap three questions of the DS14 Negative Affectivity subscale. Nevertheless, the K10 refers to a specific time period (the past four weeks) whereas the DS14 refers to the personality of the respondent as a stable trait or disposition. In fact, the prevalence of Type D personality was much higher than the prevalence of high psychological distress. Moreover, several follow-up studies of cardiac patients showed that Type D personality predicts depression, even after taking account of its baseline value [16]. In addition, the questions for Social Inhibition, an essential condition for the definition of Type D personality, do not overlap the K10.

Hence, assuming that Type D personality and low SES merely precede most health outcomes, our findings suggest that Type D personality does not explain the socioeconomic health inequalities, but is a risk factor for adverse health outcomes in addition to low SES. Moreover, for some outcomes, Type D personality even interacts with a low SES to show an excess risk.

In this community-based study, Type D personality was associated with an increased risk of adverse health outcomes, including cardiovascular disease and poor perceived physical and mental health. Furthermore, Type D personality was related to smoking but not to obesity or diabetes. Type D personality could have affected health through pathways that were not assessed in this study. For example, others have shown that Type D personality is related to lack of physical exercise [18]. Earlier findings in cardiac patients suggest that Type D personality in itself could lead to stress-related health problems due to elevated cortisol and pro-inflammatory cytokine levels, and a decreased variability of heart rate [33-37]. Another interesting finding in our study was the higher prevalence of Type D personality in the alcohol abstainers as compared to the prevalence among (un)safe drinkers. By comparison, numerous studies have also shown that alcohol abstainers (both never and former drinkers), are at greater risk of adverse health outcomes than moderate drinkers [38]. Our results might suggest that Type D personality is more related to alcohol abstinence as a risk factor for adverse health outcomes, than to unsafe alcohol use. However, a German study found that Type D personality was associated with alcohol abuse in the general population [21]. Obviously, more research is needed to clarify the role of Type D personality in the association between alcohol use and adverse health outcomes.

## Conclusions

Our results showed that the two essential conditions for the 'indirect selection hypothesis' were fulfilled: a positive association between Type D personality and low SES, as well as elevated risks of a Type D personality for most of the studied health outcomes, even conditional on a low SES. However, Type D personality did not explain the higher risks of a low SES for most (determinants of) health, as we would expect in the case of indirect selection through Type D personality, though some relative risks moved slightly towards the null value when analyzed conditionally on Type D personality.

Our findings might already be of importance for public health policies. For example, based on population attributable risks, the public health impact of Type D personality for cardiovascular disease is greater (PAR = 7.4%) than that of low education (PAR = 3.6%), though less than that of a low income (PAR = 18.5%).

Prevention in low SES populations may have more effect when it takes into consideration that persons with a low SES in combination with a Type D personality are at highest risk of adverse health outcomes and that Type D personalities, irrespective of their SES, need specific approaches, such as the diminishing of barriers for (preventive) care demand, being aware of their social fears, and improving their self-management. Acknowledging that personality is difficult to change, the main issue in prevention should probably be case finding and the tailoring of prevention programmes for this specific target group. For this, the challenge will be how to reach, identify, and influence individuals with these personalities. In the Netherlands, the general practitioner, knowing his patients, is perhaps the most appropriate person to play a pivotal role in such programmes.

## Competing interests

The authors declare that they have no competing interests.

## Authors' contributions

MvB conceived the study, conducted the statistical analyses and drafted and wrote the main part of the manuscript. JD, LK, MD, MdB and HvO provided ideas for the analysis. All authors helped draft the manuscript and read and approved the final manuscript.

## Pre-publication history

The pre-publication history for this paper can be accessed here:

http://www.biomedcentral.com/1471-2458/12/46/prepub
